# Interleukin IL-5 alleviates sepsis-induced acute lung injury by regulating the immune response in rats

**DOI:** 10.1080/21655979.2021.1930746

**Published:** 2021-05-30

**Authors:** Beichun Wei, Yu Chen, Wangmei Zhou, Xu Li, Lei Shi, Shengwu Liao

**Affiliations:** a Department of Emergency, Nanfang Hospital, Southern Medical University, Guangdong, China; b Department of Anesthesiology, The Sixth Medical Center of Chinese PLA General Hospital, Beijing, China; c Department of Burns and Plastic Surgery, The Hospital Affiliated to Jiangsu Universitity, Jiangsu, China; d School of Public Health, Southern Medical University, Guangdong, China

**Keywords:** Sepsis, acute lung injury, immune response, il-5

## Abstract

To study the effect of IL-5 on the immune response and lung injury in rats with sepsis. We constructed a rat model of sepsis by cecal ligation and puncture (CLP). The rats were randomly divided into the control group, the sham group, the CLP group and the IL-5 group, with 6 rats in each group. With the induction of CLP, the lung tissue of rats was severely injured, and the water content of lung tissue was significantly increased. Moreover, the ratio of CD4^+^/CD8^+^ was significantly decreased and Th1/Th2 was significantly increased in the peripheral blood. The content of IL-6, TNF-α, and HMGB1 was found to be increased in the CLP group. However, with the injection of IL-5, the degree of lung tissue injury in CLP rats was alleviated and the water content of lung tissue was significantly reduced. The ratio of CD4^+^/CD8^+^ was increased and Th1/Th2 was significantly down-regulated in the peripheral blood and the levels of IL-6, TNF-α, and HMGB1 in serum were significantly decreased. In conclusion, IL-5 can alleviate lung injury by regulating the immune response and inhibiting the systemic inflammatory response induced by sepsis.

## Graphical abstract

**Figure:**
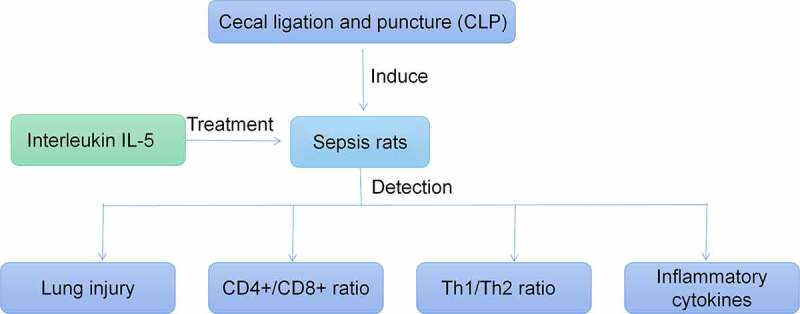

## Introduction

Sepsis is a systemic inflammatory response syndrome (SIRS) caused by infection, which involves multiple visceral organs and causes organ damage or failure [1]. Among them, lung is the initial and most vulnerable organ. There are 25% to 50% of septic patients leading to acute lung injury (ALI) [2], and the mortality rate of ALI is as high as 40% [3]. But so far there is no effective treatment and auxiliary control for sepsis and its complications. Its pathogenesis involves complex systemic inflammatory network effect, gene polymorphism, immune dysfunction, coagulation disorder, tissue damage, and abnormal host responses to different pathogenic microorganisms and their toxins [4].

The core mechanism of sepsis-induced lung injury s is the alveolar-capillary barrier dysfunction [5] caused by pulmonary or extrapulmonary infection. Inflammatory cytokines, such as IL-6, TNF-α, and HMGB1 can result in the loss of alveolar-capillary barrier integrity. Lung injury caused by the barrier dysfunction begins on the epithelial or endothelial side. After disruption of the alveolar-capillary barrier, the proinflammatory effectors of alveolar fluid are released into the circulation to promote further inflammation and immune response, neutrophil recruitment, surfactant dysfunction and alveolar edema, and thus inducing ALI [6]. Some studies have suggested that the massive release of proinflammatory cytokines and the decrease of anti-inflammatory cytokines during the inflammatory response are the fundamental mechanisms leading to ALI [7]. Therefore, regulating the release of inflammatory mediators during the inflammatory response is the key to the treatment of ALI. It has been shown that IL-5 in peripheral blood of sepsis patients decreases significantly [8]. In vivo injection of IL-33 can stimulate Th2 cells to release IL-5 and IL-13, thus improving survival rate the pathogen control in sepsis mice [9]. Another study has shown that in the treatment of idiopathic pulmonary fibrosis (IPF), increasing the content of IL-5 can reduce the mortality caused by ALI [10]. So, we speculate that IL-5 has therapeutic potential for sepsis and its complications. However, there are still no relevant studies on whether IL-5 can alleviate sepsis-induced ALI. The aim of this study is first to investigate the effect of IL-5 on ALI in sepsis rats and provide a theoretical basis for the intervention treatment of ALI in sepsis patients.

## Materials and methods

### Experimental animals and groups

A total of 24 male SD rats aged 6–8 weeks, weighing 180–220 g were randomly divided into the control group, the sham group, the CLP group and the IL-5 group, with 6 rats in each group. In the CLP group, the rats received CLP to establish the rat model of sepsis, as follows. The rats were fasted for 24 hours before the operation, but were free to obtain water. They were anesthetized by intraperitoneal injection of 10% chloral hydrate (0.3 g/kg). After general anesthesia, the abdomen was opened to expose the cecum. The cecum was ligated at 1/2, and punctured with No.21 needle in the area with the least blood vessels. A small amount of content was extruded from the perforation, and then the cecum was returned and the incision was sutured. After the operation, 30 mL/kg of normal saline was injected subcutaneously. In the sham group, the cecum was exposed and flipped without ligation and perforation. In the IL-5 group, 6 h after the model establishment, rats were injected intraperitoneally with recombinant IL-5 protein (Abcam, Cambridge, UK), and the same amount of PBS was injected at the same time in the sham group and CLP group. In the control group, the rats received no treatment.

At 48 h after the model establishment, 2 mL whole blood sample from peripheral venous blood in each group was collected using the blood collection tubes with heparin sodium, and 2 mL peripheral blood was collected using the non-anticoagulant tubes and serum was taken after centrifugation. The lung tissue was taken after euthanasia.

### Lung injury scores

One lobe of the right lung of the rats was fixed with 4% paraformaldehyde, dehydrated with ethanol and embedded in paraffin for section. The tissue was scored with the double-blind method under a 40× microscope according to the lung injury scoring criteria proposed by the American thoracic society (ATS). The scores were based on the indicators, such as alveolar congestion, hemorrhage, neutrophil infiltration or accumulation in or between vessel walls, and the thickness of hyaline membrane on the alveolar wall [11].

### Detection of the water content

The water content in the rat lung tissue was measured with dry-wet weight. The wet weight was weighed after the right lung tissue separated. Its dry weight was weighed after placed it in an incubator at 80°C for more than 48 hours until the weight no longer changed. The ratio of wet/dry weight was calculated.

### Detection of the T lymphocyte subsets in peripheral blood

The levels of CD4^+^, CD8^+^, CD4^+^/CD8^+^, and Th1/Th2 were measured using a flow cytometer (Becton, Dickinson and Company, USA). Each sample was divided into the test tube (No.1 tube) and the control tube (No.2 tube). A total of 20 μL antibody labeled by CD3^+^ FITC, CD4^+^ PerCP, CD8^+^ APC, IFN-γ PE, IL4 PE-Cy5 was added to the No.1 tube, and the No.2 tube as the isotype control. After shaking, 100 μL of anticoagulant whole blood was added and mixed, and then stained for 10–15 min in the dark. The PBS was used for rinsing once, the supernatant was aspirated, and then 0.5 mL of PBS was added to resuspend the cells. Finally, Lymphocytes are gated by forward and side scattering spectra by the flow cytometry, and the percentage of specific marker positive cells is assessed.

### ELISA to detect the levels of inflammatory cytokines and HMGB1

Interleukin-5 Assay Kit, Interleukin-6 Assay Kit, Tumor Necrosis Factor-α Assay Kit and High mobility group box 1protein (HMGB1) Kit (Nanjing Jiancheng Bioengineering Institute, China) were used to detect the content of IL-5, IL-6, TNF-α, HMGB1 in the serum of rats in each group, which were operated according to the instructions of the kits.

## Statistical analysis

SPSS26.0 was used for statistical analysis. The experimental data were expressed as mean ± standard deviation (SD). The differences between the groups were compared using the t-test, and P < 0.05 indicated statistically significant.

## Results

### IL-5 alleviates CLP-induced lung injury in rats

**Figure 1.**
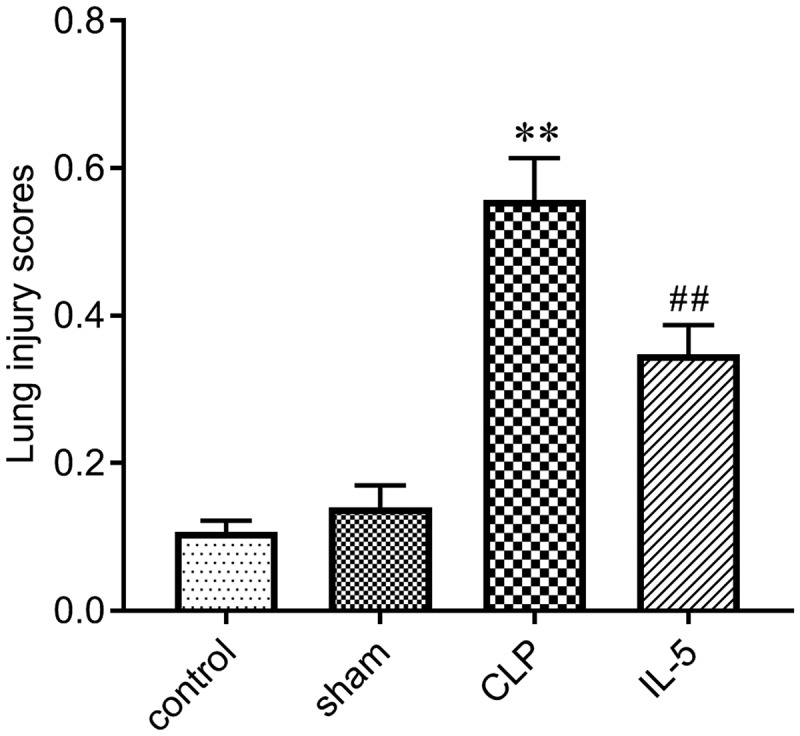
Effect of IL-5 on CLP-induced lung injury in rats

We scored the degree of lung injury to assess the protective effect of IL-5 in rats with CLP-induced lung injury. As shown in Figure 1, there was no significant difference in lung tissue damage in the Sham (0.14 ± 0.03) and Control groups (0.11 ± 0.02). The degree of lung injury in the CLP group (0.56 ± 0.06) was significantly increased compared with the sham group (P < 0.05). And the lung injury was significantly improved with the IL-5 injection (0.34 ± 0.04) (P < 0.05).

Values were expressed as mean ± SD (n = 6 in each group). **p < 0.01 vs. sham group; ^##^*p* < 0.01 vs. CLP group.

### IL-5 reduces the water content in the lung tissue of CLP rats

**Figure 2.**
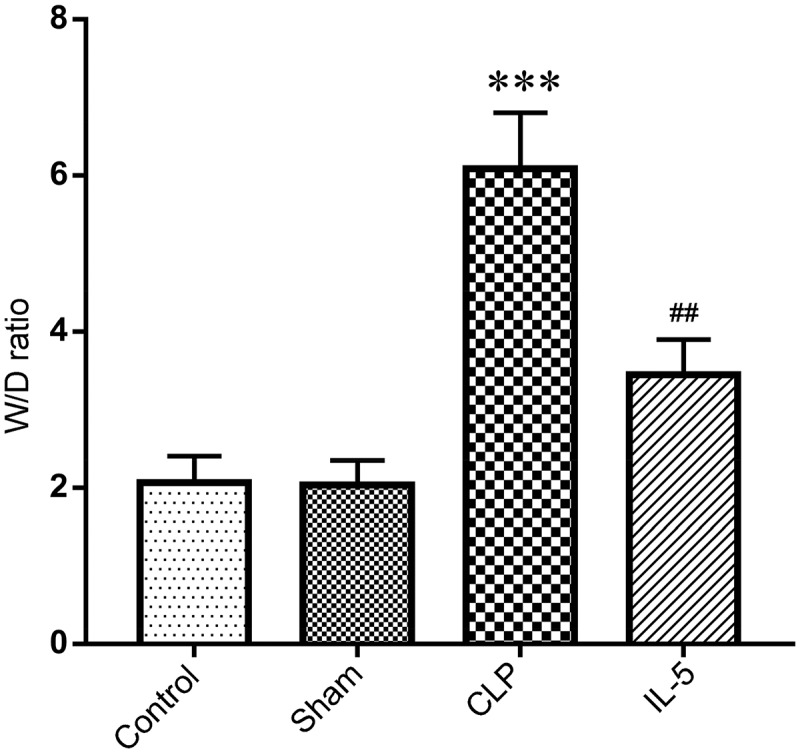
Effect of IL-5 on water content in the lung tissue of rats with CLP

The wet/dry weight ratio (W/D ratio) of lung tissue reflected its water content, that is, the degree of the pulmonary edema. We found no significant change in the water content of lung tissue in the Sham group (2.03 ± 0.32) compared with the Control group (2.06 ± 0.35). But the water content of CLP group (6.09 ± 0.72) was significantly higher than that of sham group (P < 0.05). And the water content was significantly decreased with the IL-5 injection (3.44 ± 0.45) (P < 0.05) (Figure 2).

The rats were euthanized 48 hours after CLP, and the ratio of wet (W)/dry (D) weight of the lung tissue was determined. N = 6 in each group. ***p < 0.001 vs. sham group; ^##^*p* < 0.01 vs. CLP group.

### IL-5 up-regulates the ratio of CD4^+^/CD8^+^ in the peripheral blood of CLP rats

**Figure 3.**
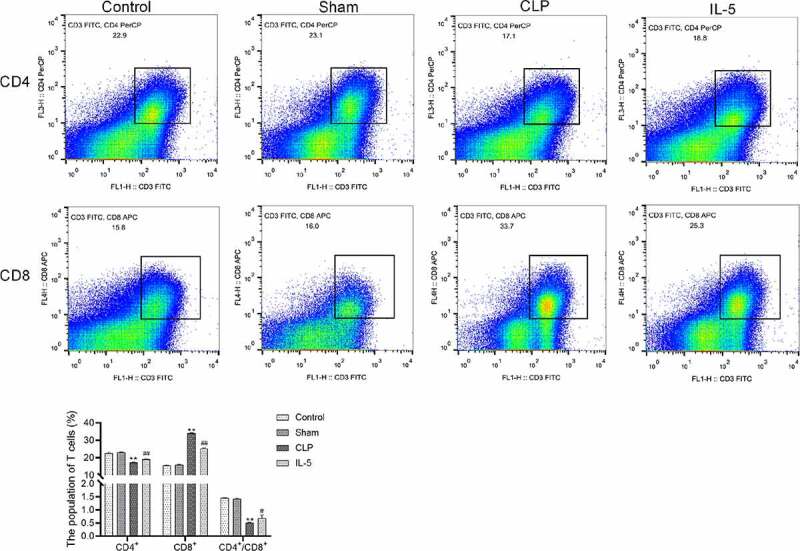
Effect of IL-5 on CD4^+^/CD8^+^ ratio in peripheral blood

The results of flow cytometry (Figure 3) showed that there was no significant difference in the proportion of each cell in peripheral blood between the sham and control groups. And compared with the sham group, the CD4^+^/CD8^+^ ratio was significantly decreased in the CLP group, with significantly decreased ratio of CD4+ and increased ratio of CD8^+^ in the peripheral blood. Compared with the CLP group, the CD4^+^/CD8^+^ ratio was significantly increased in the IL-5 group, with significantly increased ratio of CD4^+^ and significant decreased ratio of CD8^+^.

N = 3 in each group. **p < 0.01 vs. sham group; ^#^*p* < 0.05 and ^##^*p* < 0.01 vs. CLP group.

### IL-5 down-regulates the Th1/Th2 ratio in the peripheral blood of CLP rats

**Figure 4.**
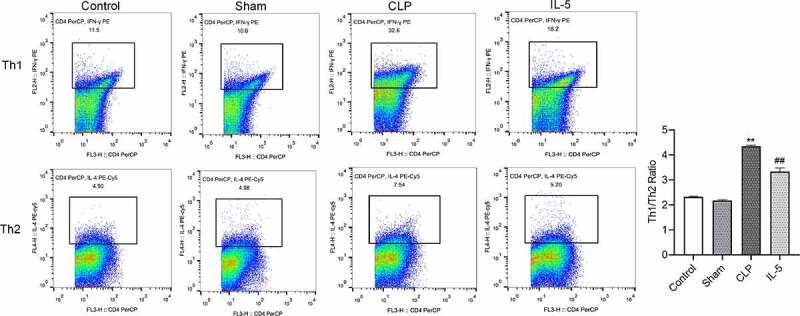
Effect of IL-5 on Th1/Th2 ratio in the peripheral blood

The results of flow cytometry (Figure 4) showed that the Th1/Th2 ratio was not significantly different between the Control and sham groups. Compared with the sham group, the Th1/Th2 ratio was significantly increased in the CLP group (P < 0.05), with significantly increased ratio of Th2 and increased ratio of Th1 in the peripheral blood. Compared with the CLP group, the Th1/Th2 ratio was significantly decreased in the CLP group (P < 0.05), with significantly decreased ratio of Th1 and decreased ratio of Th2.

N = 3 in each group. **p < 0.01 vs. sham group; ^##^*p* < 0.01 vs. CLP group.

### IL-5 decreases the expression of inflammatory cytokines in the serum of CLP rats

**Figure 5.**
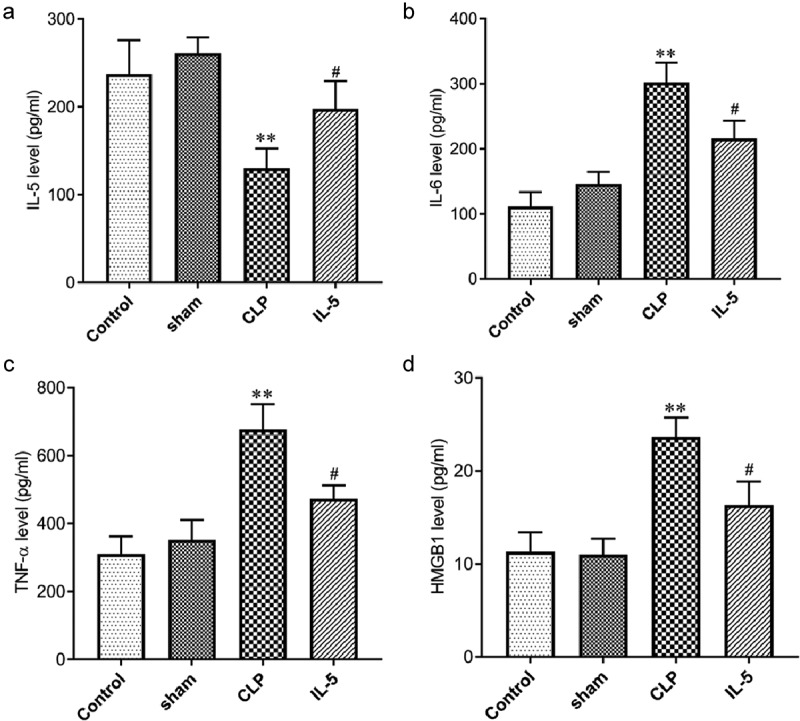
Effect of IL-5 on the content of inflammatory cytokines and HMGB1

ELISA was used to analyzed the effect of IL-5 on the content of inflammatory factors in the serum of CLP rats. As shown in Figure 5 A-D, inflammatory factor levels were not significantly different in the sham and control groups. Compared with the sham group, the IL-5 level was significantly decreased while the levels of IL-6, TNF-α, HMGB1 were significantly decreased in the CLP group (all p < 0.05). Compared with the CLP group, the IL-5 level was significantly increased while the levels of IL-6, TNF-α, HMGB1 were significantly decreased in the IL-5 group (all P < 0.05).

(A) IL-5 content in the serum in each group was detected by ELISA; (B) IL-6 content in the serum in each group was detected by ELISA; (C)TNF-α content in the serum in each group was detected by ELISA; (D) HMGB1 content in the serum in each group was detected by ELISA. N = 3 in each group. *P < 0.05 vs. sham group; * *P < 0.05 vs. sham group; # P < 0.05 vs. CLP group.

## Discussion

The main mechanisms of sepsis-induced ALI are activated neutrophil infiltration, monocyte infiltration, inflammatory cytokine disorder, pulmonary surfactant dysfunction, increased vascular permeability and others [12,13]. Among them, inflammatory cytokine disorder has been focused in recent years [14]. IL-5 is an important cytokine secreted by Th2 cells, which can target and IL-5 receptorα (IL-5 Rα), stimulate B cells growth, and increase the secretion of immunoglobulin IgA. It has been reported that IL-5 protects mice from sepsis caused by cecal ligation by targeting monocytes, so as to avoid lung injury [15]. In this study, we found that CLP induced severe lung tissue injury in rats and increased lung water content. However, the degree of the injury and the water content were significantly reduced after the injection of IL-5 in the CLP rats. And Gouda [16] and Chepurnova [17] have similar reports. However, the mechanism of IL-5 in alleviating sepsis-induced ALI has not yet been confirmed. Hrusch et al suggested that it may be that IL-5 can prevent the influx of red blood cells into the airway, and IL-5 can directly act on the structural cells of lung tissue (such as epithelium or endothelium) to reduce permeability, thus protecting the lung tissue from injury [10]. In addition, Xu [9] has shown that the reasons may be that the up-regulation of IL-5 can directly target IL-5 Rα and slow down the recruitment of active neutrophils and monocytes to the lung, thus alleviating sepsis-induced ALI. All these mechanisms need to be confirmed by in-depth studies.

Sepsis is a disorder of nonspecific immunity and specific immunity caused by infection, which leads to ALI [18]. Specific immunity inhibition mainly refers to the result of increased apoptosis of T lymphocytes (especially CD4^+^ cells) and B lymphocytes. In our study, CLP-induced sepsis significantly decreased the ratio of CD4^+^ cells, increased the ratio of CD8^+^ cells, causing a significant decrease in the ratio of CD4^+^/CD8^+^ in rats. At the same time, it significantly increased the ratio of Th1 cells, increased the ratio of Th2 cells, thus causing a significant increase in the ratio of Th1/Th2. However, the decrease of CD4^+^/CD8^+^ and the increase of Th1/Th2 were significantly alleviated with increased content of IL-5. Takamoto [19] has found that CD4^+^ cells promote each other with IL-5, while restrict each other with CD8 + . Meanwhile, IL-5 may maintain the balance of CD4^+^/CD8^+^ and Th1/Th2 ratio in the peripheral blood by reducing and the activity of Th1 and Th2 cells through negative feedback regulation [20,21]. These results are similar to our findings. Therefore, we analyze that IL-5 can control the systemic infection caused by sepsis by restoring the specific immune function.

In addition, sepsis increases the concentration of inflammatory factors (such as IL-6, TNF-α, HMGB1), which are the main inducers of apoptosis. These ligands bind to cell membrane receptors to form the complexes to induce apoptosis [22–24]. In this experiment, we found that the content of IL-6, TNF-α, HMGB1 in serum with the injection of IL-5 in CLP rats. The reason may be that IL-5 slows down the activity of T cells and monocytes [9].

## Conclusion

In summary, IL-5 can directly slow down the lung injury induced by sepsis. Besides, it also restores the balance of CD4^+^/CD8^+^ and Th1/Th2 ratio and reduces the concentrations of IL-6, TNF-α, and HMGB1, thus inhibiting the systemic inflammatory response induced by sepsis to alleviate lung injury. However, because of the complexity of the immune response, the mechanism of how IL-5 alleviates sepsis-induced lung injury by regulating immune response has not yet been well revealed. Therefore, further exploration of the relationship between IL-5 and immune response can provide a new target for the treatment of sepsis-induced ALI.

## Highlights

1. IL-5 alleviates CLP-induced lung injury in rats.
2. IL-5 reduces the water content in the lung tissue of CLP rats.
3. IL-5 increases CD4^+^/CD8^+^ ratio and decreases Th1/Th2 ratio of CLP rats.
4. IL-5 decreases the expression of inflammatory cytokines of CLP rats.
